# Comparison of the Efficacy of Two Routes of Administration of Human Amniotic Epithelial Cells in Cell Therapy of Acute Hepatic Insufficiency

**DOI:** 10.3390/ph17040476

**Published:** 2024-04-08

**Authors:** Patrycja Wieczorek, Piotr Czekaj, Mateusz Król, Edyta Bogunia, Mateusz Hermyt, Emanuel Kolanko, Jakub Toczek, Aleksandra Skubis-Sikora, Aniela Grajoszek, Rafał Stojko

**Affiliations:** 1 Department of Cytophysiology, Chair of Histology and Embryology, Faculty of Medical Sciences in Katowice, Medical University of Silesia in Katowice, Medyków 18 St., 40-752 Katowice, Poland; pszmytkowska@sum.edu.pl (P.W.); edytab@sum.edu.pl (E.B.); mhermyt@sum.edu.pl (M.H.); ekolanko@sum.edu.pl (E.K.); askubis@sum.edu.pl (A.S.-S.); 2 Department of Gynecology, Obstetrics and Oncological Gynecology, Medical University of Silesia in Katowice, Markiefki 87 St., 40-211 Katowice, Poland; streetbike5@wp.pl (J.T.); rstojko@sum.edu.pl (R.S.); 3 Department for Experimental Medicine, Medical University of Silesia in Katowice, Medyków 4 St., 40-752 Katowice, Poland; agrajoszek@sum.edu.pl

**Keywords:** acute liver failure, experimental models of hepatotoxicity, galactosoamine, stem cell therapy, human amniotic epithelial cells, cell biodistribution, intraperitoneal injection, intravenous injection

## Abstract

The route of administration of implanted cells may affect the outcome of cell therapy by directing cell migration to the damaged site. However, the question of the relationship between the route of administration, the efficacy of colonisation of a given organ, and the efficacy of cell therapy has not been resolved. The aim of the study was to localise transplanted intravenously and intraperitoneally human amniotic epithelial cells (hAECs) in the tissues of mice, both healthy and injured, in an animal experimental model of acute liver failure (ALF). Mice intoxicated with D-Galactosamine (D-GalN) at a dose of 150 mg/100 g body weight received D-GalN alone or with a single dose of hAECs administered by different routes. Subsequently, at 6, 24, and 72 h after D-GaIN administration and at 3, 21, and 69 h after hAEC administration, lungs, spleen, liver, and blood were collected from recipient mice. The degree of liver damage and regeneration was assessed based on biochemical blood parameters, histopathological evaluation (H&E staining), and immunodetection of proliferating (Ki67^+^) and apoptotic (Casp^+^) cells. The biodistribution of the administered cells was based on immunohistochemistry and the identification of human DNA. It has been shown that after intravenous administration, in both healthy and intoxicated mice, most of the transplanted hAECs were found in the lungs, while after intraperitoneal administration, they were found in the liver. We concluded that a large number of hAECs implanted in the lungs following intravenous administration can exert a therapeutic effect on the damaged liver, while the regenerative effect of intraperitoneally injected hAECs on the liver was very limited due to the relatively lower efficiency of cell engraftment.

## 1. Introduction

Acute liver failure (ALF) is caused by many factors, such as chronic use of medication (e.g., amiodarone, paracetamol), viral infections (e.g., hepatitis B virus), or diseases (e.g., Wilson’s disease) [1,2]. Despite the liver’s ability to self-regenerate from injuries induced by damaging factors, once the capacity for self-repair is exceeded, permanent and irreversible damage to the organ begins. The resulting functional disorders of the liver can lead to the sudden death of patients [3].

The significance of research on the mechanisms of hepatotoxicity and new therapies for the treatment of liver damaged by chemical agents or viruses results from the ineffectiveness of the currently used therapeutic methods, the large number of people requiring organ transplants in the absence of available donors, high treatment costs, and the lifelong need to take immunosuppressive drugs. Hepatotoxicity studies conducted on animals make it possible to analyse the mechanisms of damage, verify the efficacy of the used drugs or cells, and determine the degree of safety of their use [4,5]. However, some human liver diseases cannot be fully replicated in vivo. One example is ALF caused by the hepatitis virus, the effect of which is specific only to humans [6]. Therefore, experimental models are used in which intoxication results in symptoms that mimic human diseases. The compounds that are used to induce ALF include D-galactosamine (D-GaIN) and carbon tetrachloride (CCl_4_) [5]. Their effects vary depending on the dose administered and the frequency of administration. D-GaIN is an amino acid derivative and a highly hepatospecific toxin. The action of D-GaIN is to reduce the number of unbound uridine molecules, which prevents the synthesis of macromolecules, including RNA and protein, and causes massive necrosis of hepatocytes [7]. Just one dose of D-GaIN administered intraperitoneally can cause focal hepatocyte necrosis and a range of effects very similar to those observed in human viral hepatitis [8]. D-GaIN causes inflammatory infiltrates of histiocytes, nuclear polymorphism, dilatation of sinusoidal vessels, and the appearance of Councilman bodies [9].

In cell therapies for many diseases, attempts are made to use stem cells obtained from both adult and perinatal tissues. However, Clinicaltrials.com has only six registered projects using cell therapies to treat ALF. These studies aim to check the safety of the administered cells and determine their therapeutic properties in the treatment of damaged livers. These experiments use hepatocytes administered into the portal vein, mesenchymal stem cells isolated from bone marrow, and a commercial line isolated from adipose tissue, administered intravenously. Due to the advantages of human amniotic epithelial cells (hAECs), which have immunomodulatory properties and do not form tumours after transplantation, they are receiving increasing attention in cell therapies [10,11]. hAECs may be an efficient tool in cell therapy in the absence of an effective method of treating liver diseases. Their isolation raises no ethical concerns, and between 20 and 100 million cells can be obtained from a single amnion [12,13]. The first trials of clinical applications of hAECs showed that their administration does not cause side effects, even in newborns and adults [14,15]. Therefore, they also started to be administered to adults with liver fibrosis to establish a safe intravenous cell dose [16].

So far, it has not been possible to determine which route of hAEC administration for therapeutic purposes in ALF is safest and most efficient in the treatment of damage to the liver. It is fundamental to establish the biodistribution of transplanted cells in the recipient’s body as well as the number of implanted cells in the target organ necessary for its faster and more effective regeneration. The answer to the question of whether the implantation of transplanted cells in the damaged organ is necessary for their therapeutic effect is of great practical importance, especially since such implantation may be difficult if the degree of tissue destruction is advanced. Studies conducted on animal models as well as clinical trials show that intravenous injection is an efficient and convenient injection route for the mesenchymal stem cells (MSCs) and hAECs tested to date [16,17,18]. The direct introduction of cells into the bloodstream promotes their colonisation in the damaged organ. Compared to the above, intraperitoneal administration is faster and less invasive, but it promotes cell dispersion in the body, which may have a different impact on their therapeutic effect [19]. In addition, it is not known whether the inflammation occurring in the recipient significantly affects the biodistribution of the administered cells, promoting or not faster and more efficient cell migration to the damaged organ, and how to use this relationship to better design amniotic cell therapies. 

It was therefore hypothesised that transplanted amniotic epithelial cells may colonise the recipient’s organs in the order and time related to the route of their administration and the degree of liver damage, and the biodistribution of the administered cells in the intoxicated body may be closely related to the therapeutic effect.

The aim of this study was to compare the effectiveness of intraperitoneal and intravenous administration of isolated cells of human amniotic epithelium in colonising organs in a healthy body and one intoxicated with a hepatospecific toxin, and to assess the therapeutic effect of hAECs in an experimental animal model of ALF.

## 2. Results

### 2.1. Characteristics of Isolated hAECs

**Figure 1:**
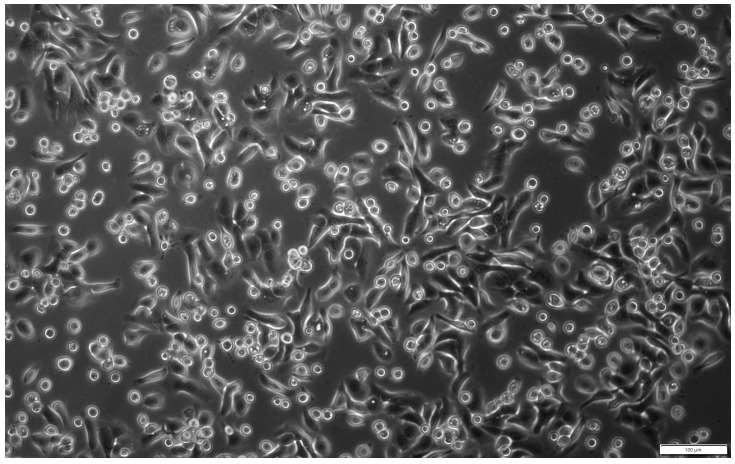
Morphology of hAECs in 24 h primary culture. The isolated cells showed adherence to the culture plate and were characterised by the typical cobblestone-like shape of epithelial cells. Magn. ×100; scale bar = 100 µm.

**Figure 2:**
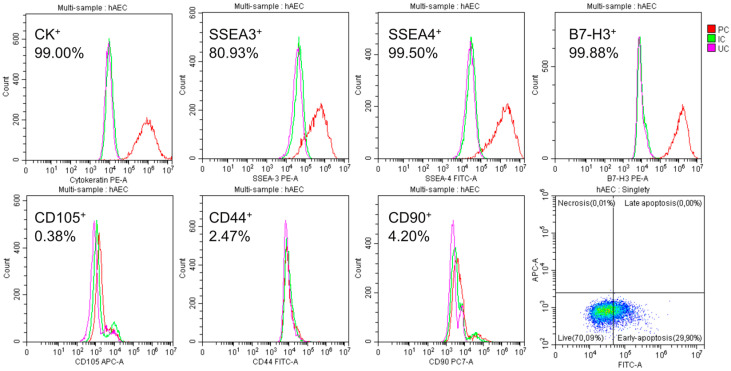
Cytometric characterization of isolated hAECs. After 48 h of culturing, large numbers of SSEA-4^+^ and SSEA-3^+^; CK14, 15, 16, and 19^+^; and B7H3^+^ were detected; and only a few CD105^+^, CD44^+^, and CD90^+^ were detected. The percentage given is the average of the six populations tested. Only a few cells showed characteristics of necrotic cells: PC—cells labelled with the corresponding antibody, IC—isotype control, and UC—cells not labelled with antibodies.

In primary culture, the obtained cells were mostly adherent and had typical epithelial cell morphology (Figure 1) and marker expression (Figure 2).

On average, 63% of the isolated amniotic cells were alive and did not show apoptotic features, 10% were in early apoptosis, and 27% were in late apoptosis. There was significant expression of the pluripotency surface markers SSEA-3 (over 80%) and SSEA-4 (almost 100%) and the immunomodulatory protein B7-H3 (almost 100%). The epithelial nature of the cells was confirmed by demonstrating the presence of the cytokeratins 14, 15, 16, and 19 and the absence of mesenchymal markers, i.e., CD105, CD44, and CD90 (Figure 2).

### 2.2. Assessment of the Degree of Liver Damage

#### 2.2.1. Assessment of Biochemical Parameters of Collected Blood

In order to assess the degree of liver damage, blood was collected at 6, 24, and 72 h after the administration of 150 mg/g bw D-GaIN and at 3, 21, and 69 h after the administration of 2 × 10^6^ hAECs. The collected blood was used to determine the activities of alanine aminotransferase (ALAT), aspartate aminotransferase (ASAT), alkaline phosphatase (ALP), and the content of total protein (TP) and glucose (Table 1).

The toxic effect of D-GalN was manifested by a statistically significant increase in ALAT and ASAT activities by 81% with a maximum at 24 h and in ALP activity from 41% to 58% between 6 and 72 h, as well as a decrease in TP content at 6 h and a significant decrease in glucose concentration at all-time points, exceeding 300% at 24 h.

The administration of hAECs to non-intoxicated mice had visible effects on the values of certain liver parameters in the blood. The effect of intraperitoneal administration was less marked than that of intravenous administration and was particularly visible in a statistically significant, approximately 60% increase in ALP activity and a significant (24–85%) decrease in glucose concentration at all-time points. For comparison, already 3 h after intravenous administration of hAECs, there was a statistically significant increase in ALAT and ASAT activities, and at 21 h also in ALP activity. These values then decreased over time. By contrast, there was a statistically insignificant (30–40%) decrease in glucose concentration, comparable at subsequent time points.

The vast majority of changes in ALAT, ASAT, and ALP activities observed after the combined administration of D-GalN and hAECs were statistically insignificant, as were changes in glucose concentration. Nevertheless, differences should be noted in the values of some parameters, especially ASAT activity and glucose concentration, between intraperitoneal and intravenous hAEC administration, amounting to several dozen percent. These changes were more severe after intravenous administration.

#### 2.2.2. Evaluation of Histopathological Changes in the Liver

Control mice showed single lymphocytes and apoptotic cells and no parenchymal damage in the liver.

**Figure 3:**
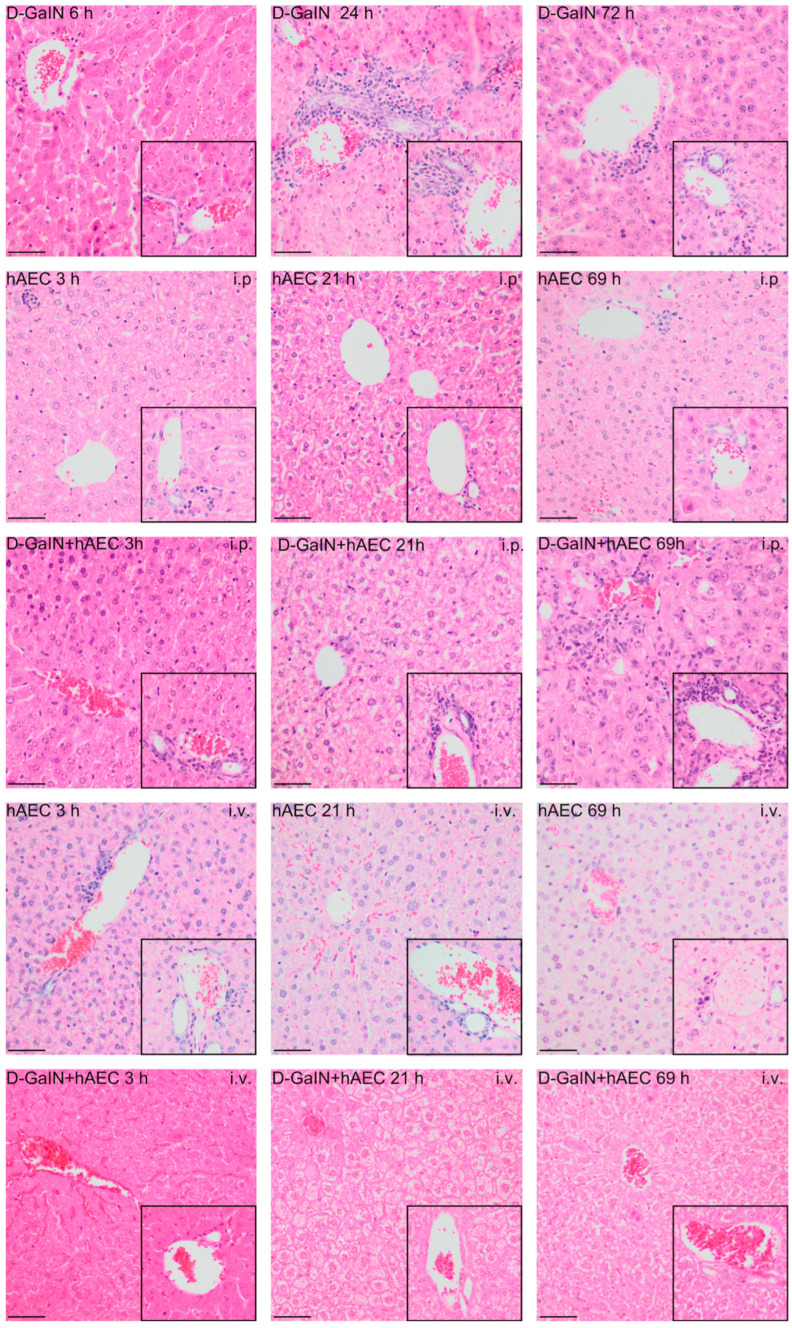
Histopathological changes in mouse livers after administration of D-GaIN and/or hAECs intraperitoneally or intravenously. The larger image shows the pericentral zone 3 and zone 2 of the hepatic acini. The inset in the lower right corner shows the area around periportal zone 1. 6/24/72 h after D-GaIN administration; 3/21/69 h after hAEC administration; i.v.—intravenous administration, i.p.—intraperitoneal administration. Control mice were injected with NaCl and showed no histopathological changes in the liver. H&E staining; Magn. ×200, scale bar = 40 µm.

**Figure 4:**
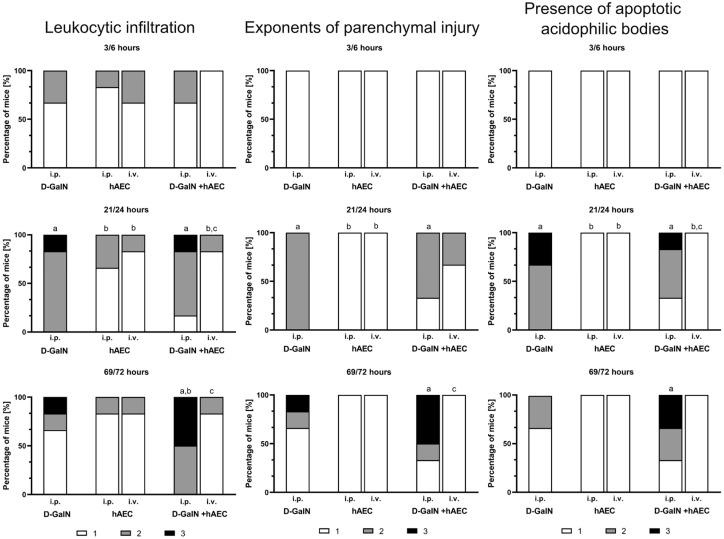
Histopathological assessment of liver damage in mice scored 1, 2, or 3 according to a three-grade histopathological classification. First number (3, 21, 69) at a given time point—time since hAEC administration; second number (6, 24, 72) at a given time point—time since D-GaIN or NaCl administration. i.p.—intraperitoneal administration; i.v.—intravenous administration; *p* < 0.05 as compared to a—the control, b—D-GaIN, c—intraperitoneal injection of hAECs. Control mice were injected with NaCl and showed no histopathological changes in the liver.

Administration of D-GaIN induced liver damage, manifested primarily by ballooning degeneration and the presence of apoptotic bodies and leukocyte clusters within hepatic acini (Figure 3). The greatest damage was recorded 24 h after intoxication, which was reflected by statistically significant differences in histopathological scores of the assessed parameters of liver damage (Figure 4). These changes decreased at 72 h after D-GaIN administration.

After both intravenous and intraperitoneal administration of hAECs, only a small number of leukocyte clusters and single apoptotic bodies were observed throughout the experiment. No signs of parenchymal damage, such as necrosis or degenerative changes in hepatocytes, were observed at any of the examined time points.

Intraperitoneal administration of hAECs after intoxication of mice increased the effects of damage observed after administration of toxin alone, manifested by a rise over time in the number of animals in which the area infiltrated by leukocytes, including degenerative changes, and the number of apoptotic cells increased. Intravenous injection of hAECs after administration of D-GaIN contributed to reducing liver damage because examination revealed only single foci of leukocytic infiltration and histopathological changes in the liver parenchyma and only very few apoptotic cells compared to the administration of D-GaIN alone and intraperitoneal administration of hAECs to intoxicated mice.

#### 2.2.3. Assessment of Liver Cell Proliferation and Apoptosis

**Figure 5:**
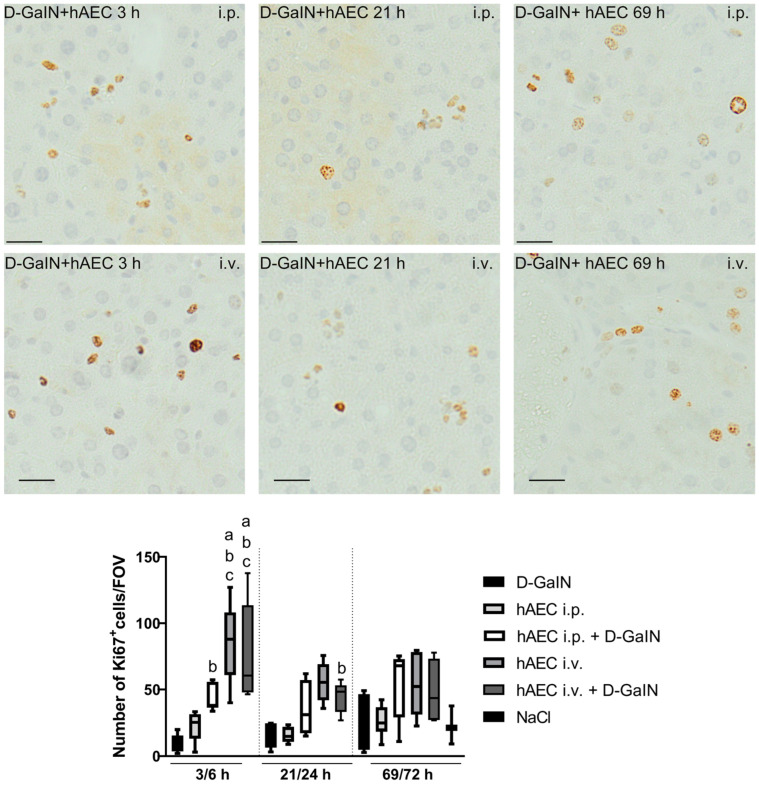
Immunodetection of proliferating cells in the liver based on the presence of Ki67 protein (**upper** images) and the number of proliferating Ki67^+^ cells in the liver expressed per cross-sectional area of 0.3779 mm^2^ (**lower** graphs) after administration of D-GaIN or NaCl (6/24/72 h) or after administration of hAECs (3/21/69 h). *p* < 0.05 as compared to a—the control, b—D-GaIN, and c—intraperitoneal injection of hAECs. Magn. ×200. Scale bar = 40 µm.

There was a variable relationship between, on the one hand, an increase in the number of proliferating cells and, on the other hand, the degree of liver damage and route of hAEC administration (Figure 5).

After the administration of D-GalN, the number of Ki67^+^ increased over time compared to the control group, but this increase was not statistically significant.

The number of proliferating cells did not increase after intraperitoneal administration of hAECs but increased statistically significantly after intravenous administration of hAECs (4-fold at 3 h and 2-fold at 21 and 69 h compared to both the control group and the group receiving hAECs intraperitoneally).

Comparing the intoxicated study groups, it was noted that just 3 h after intraperitoneal administration of hAECs, the number of proliferating cells was statistically significantly (9-fold) higher in the group receiving both hAECs and D-GaIN than in the group receiving only D-GaIN. In addition, a statistically significantly higher number of Ki67^+^ cells was observed in the groups receiving hAECs intravenously and D-GaIN compared to the group receiving only the toxin at 3 and 21 h after intoxication. These differences disappeared with increasing observation time (up to 72 h).

**Figure 6:**
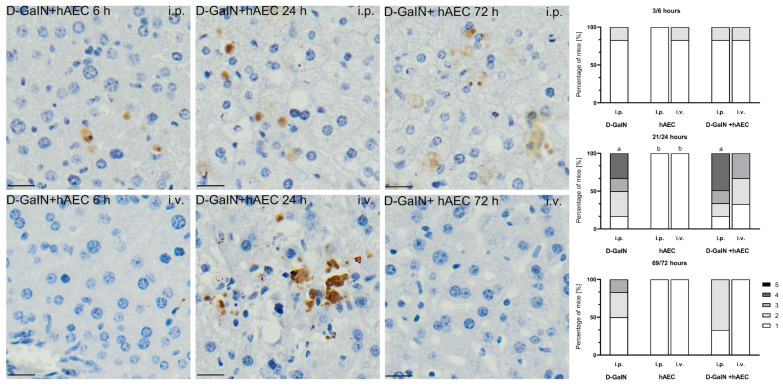
Immunodetection of apoptotic cells based on the presence of caspase-3 protein (**left** images). Percentage of mice assigned a score of 1–5 on a histopathological scale (1–5) assessing the number of apoptotic Casp^+^ cells in the liver (**right** graphs). First number (3, 21, 69) at a given time point—time since hAEC administration; second number (6, 24, 72) at a given time point—time since D-GaIN administration. *p* < 0.05 as compared to—a—the control, b—D-GaIN; i.p.—intraperitoneal administration; i.v.—intravenous administration. Magn. ×200. Scale bar = 40 µm.

After administration of D-GaIN, the number of apoptotic cells in the examined livers increased at 24 h, when it was significantly higher compared to the control group, and then decreased significantly (Figure 6).

In the groups receiving only hAECs intraperitoneally or intravenously, no apoptotic cells were detected at any of the tested time points, or very few apoptotic cells were visible.

In the groups of D-GaIN-intoxicated mice receiving hAECs, a statistically significantly higher number (by 15%) of apoptotic cells in the liver was observed at 24 h after intraperitoneal administration of cells compared to the group receiving only the toxin. In the case of intravenous administration of hAECs after previous intoxication, the number of apoptotic cells in the examined livers was lower compared to the intoxicated-only groups.

### 2.3. Identification of Transplanted Human Amniotic Cells in the Organs of Recipient Mice

**Figure 7:**
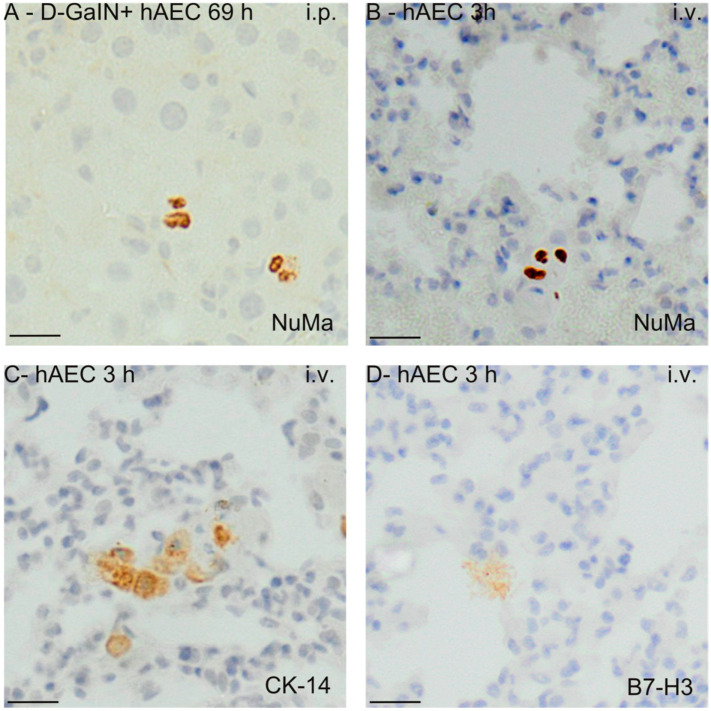
Visualisation of hAECs delivered intraperitoneally or intravenously into the mouse liver (**A**) and lungs (**B**–**D**) at the time points corresponding to the largest number of identified hAEC cells. The examples shown in the images concern immunodetection of NuMa (**A**,**B**), CK14 (**C**), and B7H3 (**D**) markers in the liver and lungs. 3/69 h—hours after hAEC administration; i.v.—intravenous administration; i.p.—intraperitoneal administration. Magn. ×200. Scale = 40 µm.

The immunohistochemical method of identifying transplanted hAECs in the liver, spleen, lungs, and blood of recipient mice involved detection of human nuclear mitotic apparatus protein (NuMA), epithelial cell marker cytokeratin 14, and human immunomodulatory protein B7-H3 (Figure 7).

**Figure 8:**
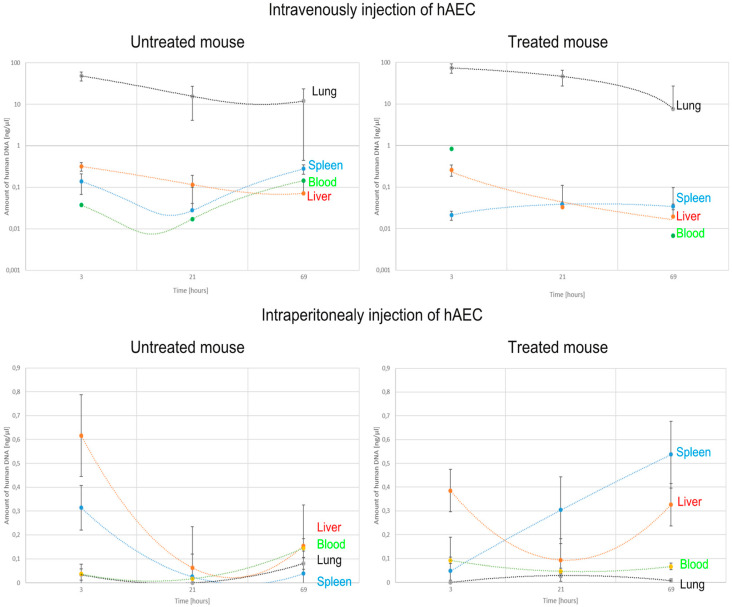
Changes in the concentration of human DNA detected in mouse organs and blood after intravenous or intraperitoneal administration of hAECs depending on the time after cell injection.

The number of implanted cells was estimated by detecting the gene encoding human cytochrome b in mice. After intraperitoneal administration to healthy mice, human DNA was present in the liver and spleen in the greatest amount 3 h after hAEC injection, and then the concentration of DNA in these organs decreased over time—in the spleen statistically significantly. In intoxicated mice, after hAEC administration, the concentration of human DNA increased significantly in the spleen and liver at 21 and 69 h. In both healthy and intoxicated mice, only small amounts of human DNA were detected in the lungs (Figure 8).

In contrast, after intravenous administration of hAECs, the largest amounts of human DNA were detected in the lungs of both intoxicated and non-intoxicated mice, but this amount decreased statistically significantly over time. Little or no human DNA was detected in the liver, spleen, and blood of these mice.

Summarising the obtained results, it can be concluded that the administration of D-galactosamine is sufficient to induce liver damage and affect the biodistribution of the administered cells. When liver damage was present, the cells administered intraperitoneally became implanted mainly in the spleen. However, when administered intravenously, they accumulated in the lungs (Table 2, Figure 8).

## 3. Discussion

Despite the many transplants performed, both from living and dead donors, the liver is still the second most-awaited organ transplant, right after kidneys [20]. The number of people requiring transplantation of damaged livers is increasing, while the number of potential donors is still insufficient. The significance of research on new alternative therapies, including stem cell therapy, for the treatment of liver damaged by chemical agents or viruses results from the ineffectiveness of the currently used therapeutic methods based on liver or hepatocyte transplantation [21,22].

The present experiment assessed the therapeutic effect of transplanted hAECs on the liver in an animal model of ALF induced by hepatospecific D-GaIN toxin. Depending on the dose and the number of injections, D-GaIN can cause liver symptoms similar to those caused by the human hepatitis virus [23,24]. Despite the well-known mechanisms of D-GaIN action on the liver, no studies have yet been conducted to test the therapeutic effect of stem cells in this model in connection with their organ biodistribution.

In the present experiment, a dose of 150 mg of D-GaIN per 100 g of body weight was used, which is believed to cause ALF but does not lead to liver necrosis [25,26]. We confirmed the efficacy of this dose by observing an increase in the values of representative blood parameters and histopathological features of hepatitis at 6 h after intoxication. The full-blown phase of ALF after D-GaIN intoxication, expressed through blood parameter values and the microscopic image of liver tissue, was observed 24 h after intoxication. At this time point, the number of apoptotic cells was the highest, which could be due to metabolization of D-GaIN and inhibition of mRNA synthesis, followed by activation of TNF-α-dependent signalling pathways [24,27]. Additionally, there was a statistically significant reduction in blood glucose levels, which may indicate that due to the competitive effect of D-GaIN against D-galactose, there was a reduction in the ability of the liver to synthesize glycogen and convert it to glucose in subsequent stages [4,5]. This would reflect the hypoglycaemic state that occurs in many ALF patients, often caused by depletion of hepatic glycogen stores and impaired gluconeogenesis [28,29]. The last phase of induced damage observed at 72 h was the regeneration phase, in which, although the features of hepatitis and degenerative changes in the parenchyma were still visible, the severity of these changes as well as the values of liver markers in the blood slowly returned to the physiological state. This was related to the inhibition of apoptotic changes and an increase in the number of proliferating cells. In total, the obtained effects of D-GalN intoxication reflect the three-stage course of action of the toxin, i.e., the initial phase visible already at 6 h after intoxication, the greatest effects of ALF at 24 h, and the self-repair processes noticeable at 72 h after intoxication [30,31].

Amniotic cells isolated from human placentas and used in the experimental model constituted a relatively homogeneous population of hAECs [10,32] characterised by the presence of epithelial cell-specific cytokeratins and the absence of markers typical of mesenchymal cells. They had some characteristics of pluripotent cells, such as the presence of the surface marker proteins SSEA-3 and SSEA-4 [33]. On average, 98% of them had the B7-H3 protein on their surface, which has a significant effect on the immune response by stimulating Th1 and Th2 lymphocytes and reducing the secretion of IL-2 and IFN-γ and the proliferation of T cells [34].

No signs of parenchymal damage, such as necrosis, were observed after either intravenous or intraperitoneal administration of hAECs to native mice. Also, no degenerative changes, signs of inflammation, or increased apoptosis were observed. In our experiment, we found no significant changes in the blood total protein concentration, although other observations indicate that after intrasplenic administration, hAECs engrafted in the mouse liver contributed to increased hepatic gene expression and secretion of normal liver proteins, including α1-antitrypsin and albumin [35,36]. Nevertheless, we recorded a significant increase in the number of proliferating cells in the liver already at 3 h after intravenous injection of hAECs, associated with a statistically significant increase in ALAT and ASAT activities, which was not observed after intraperitoneal administration of hAECs. Some substances secreted by hAECs, such as EFG and IGF, are known to have a pro-proliferative effect [37,38,39]. In addition, we noted a significant and route-independent effect of hAECs on ALP activity and blood glucose concentration. Based on preclinical studies, it is known that changes in ALP activity may be a marker of liver damage, but especially in rodents, the assessment of this activity should take into account the presence of extrahepatic forms of the enzyme, such as the intestinal isoform of ALP, showing low activity that increases, e.g., after a meal or transiently during fasting [40]. Elevated ALP activity, however, may be the result of obstruction of bile ducts caused by their blockage by the administered cells [41] and may be a manifestation of functional disorders in the liver along with changes in aminotransferase activity and increased proliferative activity of hepatocytes. Previous studies performed after administration of bone marrow cells to mice indicate that such elevated ALP activity may also reflect the role of this enzyme as a ‘signal regulator’ determining the fate of the administered cells, which was associated with the differentiation of the administered cells into osteoblasts [42]. On the other hand, the decrease in blood glucose levels observed by us in this study, particularly severe after intraperitoneal administration of hAECs, may be related to decreased appetite in injected rodents or to the passage of hAECs into the intestines and impaired glucose absorption [19,43].

The dynamics of hAEC biodistribution in healthy and intoxicated mice depended on the route of cell administration. Following intraperitoneal administration of hAECs to healthy mice, we found the presence of these cells in the blood only after 21 h, which is due to the longer time required for them to get from the peritoneum into the bloodstream and can occur in two ways. The main migration route of the administered cells is through mesenteric vessels, which carry the implanted cells to the portal vein and then to the liver. This justifies the biodistribution of the cells administered this way in the liver even before reaching the systemic circulation [44]. In addition, hAECs can enter from the peritoneal cavity into the peritoneal lymphatic vessels and only then into the bloodstream [19,45], while they can also cause clogging of small blood vessels in the liver [46,47]. The opposite situation was observed in intoxicated mice, i.e., the number of hAECs administered intraperitoneally and present in the blood increased up to 24 h and then decreased to zero at 72 h after intoxication, which coincided with increased colonisation in the spleen and, somewhat more slowly, the liver. At the same time, a small number of engrafted cells were detected in mouse lungs, both in the group receiving hAECs alone and after administration of D-GaIN and hAECs.

In general, intraperitoneal administration of hAECs was much more efficient in colonising both healthy and damaged livers. Intoxication with D-GalN, inducing histopathological changes in the liver characteristic of ALF, significantly contributed to the penetration of intraperitoneally administered hAECs into the spleen and liver. Presumably, an important role was played here by increased chemotaxis, caused mainly by the secretory activity of cells in the damaged organ [46,48,49,50]. Nevertheless, the number of cells populating the liver after intraperitoneal administration was not high enough to reveal the repair processes in the organ when assessing inflammation, histopathological changes, and the apoptotic activity of the cells. On the contrary, after the administration of hAECs, there was an increase in leukocyte infiltration, indicators of liver parenchymal damage, and the number of apoptotic cells with a simultaneous inhibition of their proliferative activity, which was hardly observed after hAEC administration to native mice.

Regarding intravenous administration of cells, it was previously shown that in healthy recipient mice, hAECs administered caudally colonise the liver as early as 1 h after implantation, but they also localise in the thoracic cavity, abdominal cavity, and hind legs [18]. We observed that after intravenous administration, the number of hAECs in the blood obviously increased at 3 h after administration, both in the group of mice receiving only hAECs and, to a greater extent, in the D-GaIN/hAEC group. In both cases, hAECs quickly disappeared from the blood, which, however, was not accompanied by an increase in their number in the liver, while there was a slight increase in their number in the spleen of intoxicated animals. The obtained results are consistent with data on MSCs, which suggested a change in the biodistribution of implanted cells when organ damage occurs [51]. However, it is known that, due to the nature of vascular connections, the lungs are potentially the most important target for migration of the cells transplanted via the intravenous route [52]. As expected, we identified hAECs in the lungs already 3 h after intravenous administration, but after 69 h, their number, although decreasing, was still significant. Similar quantitative changes in hAECs in the lungs of healthy mice were demonstrated in another study at 1, 3, and 24 h [18]. However, at 3 h after intravenous administration of hAECs to intoxicated mice, we found that the number of hAECs in the blood was higher and in the lungs was lower compared to healthy mice. This indicates that during the first hours after cell injection into intoxicated mice, only part of the administered hAECs engrafted in the lungs. Some of these cells, probably due to active attraction by chemoattractants, migrated through the circulatory system towards the site of the damage [53] but did not colonise the liver in proportionally high numbers, which can possibly be related to the toxic effect of D-GalN. Initially, the lower effectiveness of hAEC engraftment in the lungs of intoxicated mice increased at 21 h after hAEC administration, which is the time when the greatest liver damage was observed. The number of hAECs in the lungs decreased with time after intoxication, which is consistent with the results of a study on engraftment and migration of labelled MSCs administered intravenously to mice with liver necrosis, which showed that the number of cells present in the lungs decreased over time, while the number of cells engrafted in the liver and spleen remained constant and low [54]. It also appears that the cells engrafted in the lungs after intravenous administration, even if they are unable to migrate and effectively localise in the damaged organ, can produce anti-inflammatory substances into the bloodstream and have a therapeutic effect on cells of the liver damaged in the D-GaIN model already a short time after intoxication. This can take place through the secretion of anti-inflammatory substances, such as TGF-β and IL-10 [55], and substances stimulating regeneration and/or inhibiting apoptosis of hepatocytes, such as TNF-α, IL-1b, IL-2, and IL-6 [56,57], as well as through the stimulation of the proliferative activity of liver cells. The activated Kupffer cells produce substances such as TNF-α and IL-1β, which induce increased expression of E- and P-selectins on vascular endothelial cells, enabling the inflow of not only neutrophils and monocytes but also hAECs [50,58]. A paracrine therapeutic effect was also obtained in the ALF mouse model after administration of MSCs derived both from bone marrow and adipose tissue, which, despite not being engrafted in the liver, reduced the hepatotoxic effects [59,60]. Compared to intravenous administration, intraperitoneal administration of the cells should potentially favour their direct effect on the damaged liver due to their close proximity to this organ and the possibility of more intense attraction to the damage site by chemoattractants such as SDF-1. However, it was shown that in order to improve the functional efficiency of a damaged liver, the number of cells needed for its proper regeneration would have to correspond to approximately 10–15% of the organ’s weight, which enabled, among others, a reduction of blood bilirubin levels in a rat model of Crigler-Najjar type I deficiency [61,62].

The most visible effect of administering hAECs to mice previously intoxicated with D-GaIN was a significant reduction in the increases in ALAT, ASAT, and ALP activities and the decreases in glucose levels observed in mice intoxicated with D-GaIN alone. The vast majority of changes in ALAT, ASAT, and ALP activities observed after the combined administration of D-GalN and hAECs were statistically insignificant, as were changes in glucose levels. Nevertheless, differences should be noted in the values of some parameters, especially ASAT activity and glucose concentration, between intraperitoneal and intravenous hAEC administration, amounting to several dozen percent and increasing over time after the administration of hAECs. These differences indicate a decreasing toxic effect after 21 h, especially quickly after intravenous administration.

Summarising the data obtained on liver damage caused by D-GaIN administration alone and/or after hAECs administration by two routes, intravenous and intraperitoneal, we found that regardless of whether intoxication occurred or not, hAECs administered intravenously engrafted in large numbers in the lungs, while those administered intraperitoneally engrafted in the liver. Paradoxically, however, the therapeutic effect is different: it is pronounced after intravenous administration, most likely due to the large number of engrafted cells and increased concentration of factors secreted by the administered hAECs, while after intraperitoneal administration, it does not occur and the inflammatory and degenerative changes intensify, which may be due to the insufficient effectiveness of the engraftment of hAECs in the damaged organ.

## 4. Materials and Methods

### 4.1. Experimental Model of Hepatotoxicity

The study was conducted on adult, eight-week-old female BALB/c mice obtained from the Centre for Experimental Medicine at the Medical University of Silesia in Katowice. During the experiment, the animals were kept in separate cages under standard conditions: temperature of 22 °C ± 2 °C, humidity of 50–60%, light/dark cycle of 12 h/12 h, and light intensity of 60–400 lux. Water and food (Labofeed) were available ad libitum.

Healthy mice were divided into four main groups of six mice each, receiving single injections of placebo (group I—intraperitoneally, group II—intravenously), hAECs administered intraperitoneally (group III), and hAECs administered intravenously (group IV), respectively.

Mice subjected to intoxication received D-GalN by intraperitoneal injection at a single dose of 150 mg/100 g bw. The injection contained galactosamine hydrochloride solution with pH = 6.8 equilibrated with NaOH solution, administered using disposable sterile insulin syringes.

Mice injected with D-GalN were divided into three subgroups: one received D-GaIN alone (group V), the second also received hAECs intraperitoneally (group VI), and the third also received hAECs intravenously (group VII) (Table 3). 

**Figure 9:**
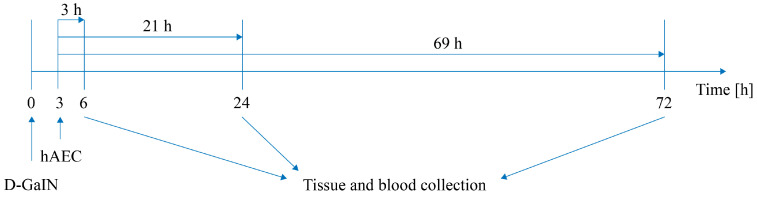
Scheme of the experiment. Intoxication time precedes hAEC administration by 3 h.

Subsequently, at 6, 24, and 72 h after D-GaIN administration and at 3, 21, and 69 h after hAEC administration, lungs, spleen, liver, and blood were collected from recipient mice (Figure 9). hAECs were visualised in the collected tissues and blood by immunohistochemistry and identified by the detection of human DNA. In addition, biochemical and morphological testing of mouse blood was performed to assess recipient health and liver damage, and histological staining with haematoxylin and eosin was used to assess histopathological changes induced in the liver.

### 4.2. Isolation of hAECs

Human placentas were collected from women aged 18–35 who were patients of the Department of Gynaecology and Obstetrics with the Division of Gynaecologic Oncology at the Brothers Hospitallers Hospital in Katowice after obtaining their informed consent. The placentas came from normal pregnancies terminated at full term by caesarean section performed for obstetric reasons (transverse or longitudinal pelvic position of the foetus, foetopelvic disproportion) or for non-obstetric indications in the absence of symptoms of placental insufficiency. The study was performed on cells isolated from six placentas with the approval of the Bioethics Committee at the Medical University of Silesia in Katowice (permission no. KNW/0022/KB/29C/19).

Inclusion criteria: good health, uncomplicated pregnancy, and full-term delivery.

Exclusion criteria: smoking, drinking alcohol or use of other stimulants during pregnancy, positive test for group B streptococcus, and chronic non-obstetric diseases prior to or during pregnancy.

The placentas were collected in a sterile container containing phosphate buffer (PBS) supplemented with an anticoagulant agent (5 mM EDTA) and an antibiotic–antimycotic solution (penicillin 0.1 U/mL, streptomycin sulphate 0.1 mg/mL, and amphotericin B 0.25 μg/mL). The amniotic membrane was mechanically separated from the chorion, after which fragments measuring approximately 1 cm × 1 cm were cut off from the membrane and decellularized by enzymatic digestion with trypsin (0.05%, 40 min, 37 °C). The cell suspension obtained after digestion was centrifuged (Eppendorf Centrifuge 5810R, at 500× *g*, 5 min, 4 °C) and suspended in culture medium (DMEM, 10% FBS, 1% AA, 10 ng/μL EGF). The hAECs were cultured in an incubator under standard conditions, i.e., 37 °C and 5% CO_2_. The cells were seeded at a density of 160,000/cm^2^ in a 25 cm^2^ culture flask. After 48 h of culture, cell viability was assessed, and the phenotype was characterised. The remaining isolated cells were banked.

### 4.3. Assessment of Viability and Phenotype of Isolated Amniotic Cells

After 48 h of culturing isolated cells, the phenotype was analysed by detection of the following markers: pluripotency markers SSEA-3 (PE Rat anti-SSEA3 cat. No. 560237; BD Biosciences, Franklin Lakes, NJ, USA) and SSEA-4 (FITC Mouse anti-SSEA-4, cat. no. 560126; BD Biosciences, Franklin Lakes, NJ, USA); the immunomodulatory protein B7H3 (PE anti-B7-H3 (CD276), cat. no. FAB1027P (R&D Systems, Minneapolis, MN, USA); cytokeratins CK14, CK15, CK16, and CK19 (Kit PE Mouse Anti-Human Cytokeratin 14/15/16/19, cat. no. 550953; BD Biosciences, Franklin Lakes, NJ, USA); and mesenchymal markers CD105, CD44, and CD90 (FITC Mouse Anti-Human CD44, cat. no. 555478; Alexa Fluor 647 Mouse anti-Human CD105, cat. no. 561439; PE-Cy7 Mouse Anti-Human CD90, cat. no. 561558, BD Biosciences, Franklin Lakes, NJ, USA). To evaluate the phenotype of the obtained cells, after digestion from the culture flask with TrypLE Express enzyme solution (Life Technologies, Thermo Fisher Scientific, Waltham, MA, USA), hAECs in the amount of 1 × 10^6^ were suspended in culture medium, centrifuged (500× *g*, 5 min, 4 °C), resuspended in a staining solution (PBS supplemented with 10% FBS and 5 mM EDTA) containing fluorochrome-conjugated antibodies or appropriate isotype controls, and incubated at room temperature for 30 min. After washing the cell suspension in the staining solution and centrifuging (500× *g*, 5 min, 4 °C), the cells were resuspended in the staining solution and analysed in a flow cytometer (CytoFlex; Beckman Coulter, Brea, CA, USA). Cytometric analysis was performed after 10,000 cells were counted.

To assess cell viability and apoptotic cell content in the culture, after digestion from the culture flask, 100,000 cells were resuspended in 1 x concentrated binding solution from the manufacturer’s kit (FITC Annexin V Apoptosis Detection Kit I, cat. no. 556547, BD Biosciences, Franklin Lakes, NJ, USA) and labelled with propidium iodide and Annexin V solution. The cells were then incubated with the antibody for 15 min at room temperature in the dark. In the final step, the labelled cell suspension was diluted fivefold to inhibit the reaction and analysed in a flow cytometer (CytoFlex; Beckman Coulter, Brea, CA, USA). Cytometric analysis was performed after 10,000 cells had been counted.

### 4.4. Delivery of Isolated hAECs

Forty-eight hours before the planned administration, hAECs were thawed, seeded in culture flasks, and cultured in an incubator (Sanyo MCO-19M, Osaka, Japan) under standard conditions, i.e., 37 °C and 5% CO_2_ concentration. After a predetermined culture time, the cells were digested from the surface of the culture flask with TrypLE Express enzyme solution (Thermo Fisher Scientific, Waltham, MA, USA), and the obtained 2 × 10^6^ hAECs were suspended in 0.9% NaCl and administered to mice intraperitoneally or into the caudal vein. The cells were administered in a single dose using a 27 G 1/2-inch (0.4 × 14) insulin needle syringe (BD). The mice in the control group received 250 µL of saline.

### 4.5. Evaluation of the Liver Damage 

For histopathological and immunohistochemical evaluation, the liver, spleen, lungs, and blood were harvested from mice euthanized 6, 24, and 72 h after D-GaIN administration and 3, 21, and 69 h after hAEC administration.

#### 4.5.1. Blood Parameters in the Recipient Mice

Blood tests were performed at Labo-Vet (Czechowice-Dziedzice, Poland) 6, 24, and 72 h after administration of D-GaIN and 3, 21, and 69 h after administration of hAECs or 72 h after administration of NaCl. Haematological tests were performed on an ABX Micros ESV60 haematology analyser using a combination of two measurement methods: impedance and photometry. Biochemical blood tests (ALAT, ASAT, ALP, total protein, and glucose) were performed using an Accent-200 analyser, which measures the absorbance of the reaction mixture.

#### 4.5.2. Histopathological Evaluation

In order to standardise the assessment, a three-grade classification of liver damage markers was introduced (Table 4).

#### 4.5.3. Assessment of Liver Damage Based on Histological Staining with Haematoxylin and Eosin

The tissues were fixed in 10% buffered formalin and then embedded in paraffin. Paraffin blocks were cut on a Microm HM 350 S microtome (Thermo Fisher Scientific, Waltham, MA, USA) into 4-μm-thick sections, which were deparaffinised in xylene and decreasing concentrations of alcohols and then stained with haematoxylin and eosin (H&E) or stained immunohistochemically. The degree of liver damage was assessed after H&E staining by the presence of the following markers: interface hepatitis, parenchymal liver damage, and the presence of acidophilic bodies.

#### 4.5.4. Evaluation of Proliferative and Apoptotic Activity of Hepatic Cells

Paraffin-embedded sections obtained from mouse livers, after deparaffinisation and rehydration, were immunohistochemically stained to detect Ki-67, a marker of proliferation, and caspase-3, a marker of apoptosis. For this purpose, specific antibodies and isotype controls were used, as well as a positive control, which was a section prepared from a human tonsil. The unmasking of Ki-67 antigen and caspase-3 was performed using a citric acid solution (Vector, Newark, CA, USA) by incubation for 60 min. To eliminate non-specific binding, the prepared liver sections were incubated for 60 min in a 2.5% horse serum solution (Vector, Newark, CA, USA). The sections were then incubated with either rabbit anti-Ki67 antibody (ab16667; dilution 1:400, Abcam, Cambridge, UK) or rabbit anti-caspase 3 antibody (#9661; dilution 1:500; Cell Signalling, Danvers, MA, USA) for 20 h at 4 °C, and with a peroxidase-conjugated secondary anti-rabbit antibody (ImPreSS Vector Laboratories, Newark, CA, USA) for 30 min at room temperature. The staining reaction was carried out using 0.05% diaminobenzidine (Vector, Newark, CA, USA). The sections were then stained with haematoxylin to visualise cell nuclei, dehydrated in a series of increasing concentrations of alcohols and xylene, and sealed with a coverslip in DPX medium. To assess the number of positive cells on each section, fifteen random fields of 0.3779 mm^2^ each were photographed at 100× magnification. Ki67 immunodetection results were analysed using ImageJ 1.52e software and expressed as the average number of positive cells present per area. A semi-quantitative ordinal scale was used to assess the abundance of Casp^+^ apoptotic cells expressing caspase-3, namely: (1) no Casp^+^ cells in section; (2) <10 Casp^+^ cells; (3) 10–25 Casp^+^ cells; (4) 26–50 Casp^+^ cells; and (5) >50 Casp^+^ cells per area (0.3779 mm^2^) (Table 5). 

### 4.6. Identification of Transplanted Human Amniotic Cells in Recipient Mouse Tissues

#### 4.6.1. Immunohistochemical Detection of hAECs

hAECs were identified in paraffin sections obtained from mouse lungs, liver, and spleen collected at 6, 24, and 72 h after intoxication or at 3, 21, and 69 h after hAEC administration. Detection of hAECs in mouse organs was carried out by identifying human proteins, i.e., the nuclear protein of the mitotic apparatus NuMa, the immunomodulatory protein B7-H3, and the intermediate filament protein cytokeratin 14. For this purpose, deparaffinised sections were incubated with a 2.5% goat serum solution to eliminate non-specific binding of primary antibodies. The sections were incubated with the appropriate primary antibody (Rabbit Anti-NuMA antibody, cat. no. ab84680; Rabbit Anti-B7H3 antibody, cat. no. ab105922; Rabbit Recombinant Anti-Cytokeratin 14 antibody, cat. no. ab51054; all from Abcam, Cambridge, UK) or isotypic control for 20 h at 4 °C. This was followed by a 30 min incubation with a peroxidase-conjugated anti-rabbit secondary antibody (ImmPress Vector, Newark, CA, USA). Visualisation was achieved with 0.05% diaminobenzidine (Vector Laboratories), after which the sections were stained with haematoxylin to visualise cell nuclei, dehydrated in a series of increasing concentrations of alcohols and xylene, and sealed with a coverslip in DPX medium.

#### 4.6.2. Identification of Human DNA from Transplanted hAECs

The presence of human amniotic cells was assessed in recipient mouse tissues and blood by identifying the human mitochondrial cytochrome b gene. Total DNA was extracted from mouse tissues using the DNA/RNA Extracol Kit (EurX, Gdańsk, Poland) and from blood using the Quick Blood DNA Purification Kit according to the manufacturer’s instructions. The amplification reaction was carried out on a Roche LightCycler 480, and the specificity of the resulting product was confirmed by agarose gel electrophoresis and the determination of the melting temperature (Tm) curve. For the detection of cytochrome b, the FastStart Essential DNA Green Master reagent kit (Roche, Indianapolis, USA) and oligonucleotide primers (Thermo Fisher Scientific, Waltham, MA, USA) specific for the tested gene, were used (Table 6).

The concentration of human DNA, as detected by cytochrome b sequencing, was determined using a standard DNA concentration curve (TaqMan DNA Template Reagents, Thermo Fisher Scientific, Waltham, MA, USA) and compared with the concentrations obtained from a known number of isolated hAECs, i.e., 1000, 5000, 10,000, 100,000, 250,000, 500,000, 1 million, and 1.5 million. Using the equation defining the relationship between the concentration of human DNA and the CT value obtained from 100 ng of the DNA of the tested mouse tissue, the concentration obtained per 1 mg of DNA isolated from the tested tissues was calculated so that it was possible to compare the content of human DNA in samples with different concentrations of starting DNA. A comparison of the obtained concentration of human DNA from a given number of hAECs to the data from the standard curve allowed a preliminary estimate of the number of implanted cells in mouse organs.

### 4.7. Statistical Analysis

Statistical analysis was performed using Statistica 13.3 software (TIBCO, USA). Different groups were compared using Kruskal–Wallis test for non-normally distributed data and an ANOVA with a post hoc Tukey test for normally distributed data. The level of significance was set at *p* < 0.05 for all statistical tests. Values were expressed as median (Me) with the 25th and 75th quartiles and minimum and maximum for non-normally distributed data, and for normally distributed variables, they were presented as mean and standard deviation.

## 5. Conclusions

1. The biodistribution of transplanted hAECs varies depending on the route of administration and the degree of liver damage.
2. After intravenous administration, most of the cells engraft in the lungs, while after intraperitoneal administration, they engraft primarily in the liver in both intoxicated and native mice.
3. The increased presence of hAECs in the spleen is associated with progressive liver toxicity regardless of the route of administration—the greater the liver damage, the greater the number of hAECs in the spleen.
4. After intravenous administration, a large number of the grafted hAECs in the lungs can have a therapeutic effect on the damaged liver.
5. The therapeutic effect of intraperitoneally administered hAECs on histopathological and regenerative changes in the liver is very limited due to the low efficiency of cell engraftment in the damaged organ.

## Figures and Tables

**Table 1:** Changes in blood parameters after administration of D-GaIN, and/or hAECs depending on the route of cell injection. Statistically significant (*p* < 0.05) as compared to the: a—control; b—D-GaIN.

Intoxication/Cell Delivery	Time after Injection	Route of hAEC Administration	ALAT	ASAT	ALP	TP	Glucose
D-GaIN	6 h	intraperitoneally	9% ↑	18% ↑	58% ^a^↑	18% ^a^↓	89% ^a^↓
	24 h		81% ^a^↑	81% ^a^↑	56% ^a^↑	1% ↓	329% ^a^↓
	72 h		76% ^a^↑	46% ^a^↑	41% ^a^↑	7% ↓	170% ^a^↓
hAEC	3 h	intraperitoneally	37% ↑	8% ↑	59% ^a^↑	0%	51% ^a^↓
		intravenously	61% ^a^↑	82% ^a^↑	52% ↑	2% ↓	32% ↓
	21 h	intraperitoneally	9% ↑	12% ↑	63% ^a^↑	2% ↓	24% ^a^↓
		intravenously	56%↑	55% ^a^↑	49% ^a^↑	3% ↓	37% ↓
	69 h	intraperitoneally	6% ↑	13% ↓	62% ^a^↑	7% ↑	85% ^a^↓
		intravenously	11%↓	38% ↑	21% ↑	3% ↑	41% ↓
hAEC + D-GaIN	3/6 h	intraperitoneally	27% ↑	6% ↑	7% ↑	10% ^b^↑	0%
		intravenously	20% ↑	39% ↑	13% ↓	9% ↑	38% ↓
	21/24 h	intraperitoneally	41% ↑	16% ↑	6% ↓	7% ↓	6% ↓
		intravenously	31% ↓	29% ↑	3% ↓	0%	34% ↑
	69/72 h	intraperitoneally	65% ↑	40% ↑	15% ↑	6% ↑	11% ↑
		intravenously	443% ^b^↓	89% ↓	38% ↓	15% ^b^↑	36% ^b^↑

**Table 2:** Number of hAECs estimated from the concentration of human DNA in intoxicated and untreated mouse lungs, spleen, liver, and blood, depending on the route of cell administration. Values are medians of hAECs. *p* < 0.05 as compared to—a—intraperitoneal injection of hAEC, b—3 h in untreated mice; c—3 h in treated mice; d—21 h in untreated mice; e—21 h in treated mice; f—69 h in untreated mice (n = 6).

Organ	Route of hAEC Administration	Untreated Mouse			Treated Mouse		
		Time [h]					
		3	21	69	3	21	69
Lung	Intraperitoneally	2	0 ^b^	5 ^b,d^	0	0	0
	Intravenously	138,397 ^a^	23,581 ^a,b^	19,400 ^a,b^	23,496 ^a^	77,361 ^a^	11,450 ^a^
Spleen	Intraperitoneally	332	0 ^b^	92	69	788 ^c,d^	361 ^c,f^
	Intravenously	0	5	0	0	71 ^a^	55 ^a^
Liver	Intraperitoneally	449	125	343	261	139	552 ^e^
	Intravenously	189	0 ^b^	59 ^a^	22 ^a^	0 ^a^	0 ^a^
Blood	Intraperitoneally	0	11 ^b^	8 ^b^	22	33	0
	Intravenously	18 ^a^	0	0	905 ^a^	0 ^a,c^	0

**Table 3:** Description of the study groups.

Group		Subgroups	Route of Administration	Injection
Control group	I	72O	Intraperitoneally	NaCl
	II	72Z	Intravenously	
hAEC injection group	III	3O	Intraperitoneally	hAEC
		21O		
		69O		
	IV	3Z	Intravenously	
		21Z		
		69Z		
D-GaIN injection group		6G	Intraperitoneally	D-GaIN
	V	24G		
		72G		
D-GaIN and hAEC injection group	VI	6GO	D-GaIN intraperitoneally; hAEC intraperitoneally	D-GaIN and hAEC after 3 h
		24GO		
		72GO		
	VII	6GZ	D-GaIN intraperitoneally; hAEC intravenously	
		24GZ		
		72GZ		

**Table 4:** Histopathological scale to assess the degree of liver damage.

Scale	Hepatitis Exponents	Scale	Exponents of Liver Parenchymal Injury	Scale	Presence of Acidophilic Apoptotic Bodies
1	No or minimal inflammation; absent or single infiltration in zone 1 of the hepatic stroma; or single infiltration of the parenchyma	1	No foci of damage/single foci of damage; parenchymal oedema	1	None
2	Infiltration in zone 1 of the hepatic stroma occupying <50% of the examined area	2	At least one outbreak of damage	2	Singular
3	Infiltration in zone 1 of the hepatic stroma occupying >50% of the examined area	3	Multiple foci of damage; necrosis; disruption of parenchymal architecture	3	Multiple

**Table 5:** Scale for assessing the number of Casp^+^ apoptotic cells in recipient mouse liver sections per surface area (0.3779 mm^2^).

Scale	Evaluation
1	No protein expression
2	Protein present in <10 cells examined
3	Protein present in 10–25 cells tested
4	Protein present in 26–50 cells tested
5	Protein present in >50 cells tested

**Table 6:** The primers used for the qPCR reaction.

Gene	Name	Sequence	Product Length	Tm [°C]
CB	Cytochrome b	F: 5′-CCCATACATTGGGACAGACC-3′ R: 5′-GACGGATCGGAGAATTGTGT-3′	394	82.5

## Data Availability

Data are contained within the article.
